# Rapid Discharge After Interfacility Transfer for Mild Traumatic Intracranial Hemorrhage: Frequency and Associated Factors

**DOI:** 10.5811/westjem.2018.12.39337

**Published:** 2019-02-11

**Authors:** Pierre Borczuk, Jonathan Van Ornam, Brian J. Yun, Joshua Penn, Peter Pruitt

**Affiliations:** * Harvard Medical School, Massachusetts General Hospital, Department of Emergency Medicine, Boston, Massachusetts; † Winchester Hospital, Emergency Services, Winchester, Massachusetts; ‡ Northwestern University, Department of Emergency Medicine, Chicago, Illinois

## Abstract

**Introduction:**

Traumatic intracranial hemorrhage (TIH), brain injury with radiographic hemorrhage, is a common emergency department (ED) presentation, and encompasses a wide range of clinical syndromes. Patients with moderate and severe neurotrauma (Glasgow Coma Scale [GCS] < 13) with intracranial hemorrhage require care at a trauma center with neurosurgical capabilities. However, many patients with mild traumatic intracranial hemorrhage (mTIH), defined as radiographic bleeding and GCS ≥ 13, do not require operative intervention or intensive care unit monitoring, but are still routinely transferred to tertiary care centers. We hypothesized that a significant proportion of patients are managed non-operatively and are discharged within 24 hours of admission.

**Methods:**

This was a retrospective, observational study of consecutive patients age ≥ 16 years, GCS ≥ 13 who were transferred to an urban, medical school-affiliated, 100,000 annual visit ED over a seven-year period with blunt isolated mTIH. The primary outcome was discharge within 24 hours of admission. We measured rates of neurosurgical intervention, computed tomography hemorrhage progression, and neurologic deterioration as well as other demographic and clinical variables.

**Results:**

There were 1079 transferred patients with isolated mTIH. Of these, 92.4% were treated non-operatively and 35.8% were discharged within 24 hours of presentation to the tertiary ED. Patient characteristics associated with rapid discharge after transfer include a GCS of 15 (odds ratio [OR] 2.9, 95% confidence interval [CI], 1.9 – 4.4), subdural hematoma ≤ 6mm (OR 3.1, 95% CI, 2.2 – 4.5) or the presence of an isolated subarachnoid hemorrhage (OR 1.7, 95% CI, 1.3 – 2.4). Of patients with length of stay < 24 hours, 79.8% were discharged directly from the ED or ED observation unit.

**Conclusion:**

Patients transferred to tertiary care centers are frequently discharged after brief observation without intervention. Risk can be predicted by clinical and radiographic data. Further prospective research is required to determine a safe cohort of patients who could be managed at community sites.

## INTRODUCTION

Traumatic intracranial hemorrhage (TIH) is common and encompasses a wide variety of clinical syndromes. There were an estimated 2.5 million emergency department (ED) visits for traumatic brain injury (TBI) in 2010, with the rate of TBI visits having increased eight-fold more than the rate of total ED visits during that time.^1^ Patients with TIH have TBI with an associated radiographic finding, usually on computed tomography (CT) imaging. Multiple different types of lesions, including subdural hematoma (SDH), traumatic subarachnoid hemorrhage (SAH), cerebral contusions and epidural hematomas, make up the broader group of TIH. Importantly, each of these disease subtypes has a unique clinical trajectory, which depends on both the type of lesion and the severity of injury at presentation.^2^,^3^

In organized trauma systems, patients are routinely transferred to tertiary care centers. Trauma systems reduce mortality for patients with severe injuries^4^ by providing these critically ill patients rapid access to specialized physicians and care environments.^5^ In the hub-and-spoke model of trauma care, emergency medical services protocols aid in the primary triage of injured patients to a local hospital (spoke) vs direct transportation to a Level I trauma center (hub). Emergency physicians or trauma surgeons at spoke sites must determine which patients have severe injuries or require specialized consultation that warrants transfer to the hub. This transfer decision is not as rigorously defined as those for the prehospital providers. Advanced Trauma Life Support provides some guidance, but lack of routine protocols means that much is done based on physician gestalt and historic clinical practice.

While validated guidelines exist to aid the decision to image patients with head trauma,^6^ the subsequent disposition of patients who are diagnosed with TIH is not as clear. There is ample evidence to support the transport of individuals with severe and moderate TBI (Glasgow Coma Scale Score [GCS] <12)^7^,^8^ to a trauma center for evaluation by a neurosurgeon and management in an intensive care unit (ICU).^9^ Clinicians extrapolate this transfer practice and also routinely transfer patients with mild traumatic intracranial hemorrhage (mTIH), defined as having intracranial hemorrhage (IH) with a GCS of 13–15, though there is growing evidence that some patients with mTIH may be able to be managed in lower resource settings.^2^,^10^–^12^ However, a relative lack of resources, including advanced monitoring capabilities or neurosurgical backup, as well as clinical experience, may make community providers understandably hesitant to defer transfer and monitor these patients at the presenting facility.

Rapid discharge after transfer (RDAT) is a relatively common phenomenon.^13^–^15^ In one study over a two-year period at a Level I trauma center, 24% of patients transferred after injury were discharged within 24 hours of admission; orthopedic injuries and head trauma were the top two organ systems in patients found to be quickly discharged.^16^ There has been no prior investigation evaluating RDAT solely in patients with mTIH. Given the frequency with which these patients are transferred and their heterogeneous clinical prognosis, this is likely a population in which a better understanding of risk could be used to streamline care processes.

This study attempted to quantify the frequency of RDAT in a retrospective cohort of patients. We hypothesized that a substantial percentage of patients transferred to the tertiary facility would be non-operatively managed and discharged within 24 hours of their transfer. Our secondary objective was to identify clinical and radiographic factors associated with RDAT. Additionally, we explored the association of these factors with other clinical endpoints, including death, neurosurgical intervention and evidence of worsening on CT imaging.

**Population Health Research Capsule**

What do we already know about this issue?

Most patients with traumatic intracranial hemorrhage (TIH) and Glasgow Coma Scale Score ≥ 13 transferred for neurosurgical consultation do not require operative intervention or intensive care unit admission.

What was the research question?

What percentage of patients with mild traumatic intracranial hemorrhage (mTIH) are discharged within 24 hours post neurosurgical evaluation/observation?

What was the major finding of the study?

Of 1079 transferred patients with isolated mTIH, 35.8% were discharged within 24 hours of emergency department presentation.

How does this improve population health?

Streamlining mTIH care by improving use of community hospital resources can concurrently unburden trauma centers, decrease costs, and reduce family inconvenience.

## METHODS

### Study Design, Setting and Participants

This was a retrospective, observational study performed at a single urban, academic Level I trauma center with an annual ED volume of over 100,000 visits. Patients age ≥16 with blunt head trauma were identified by running a query of a proprietary electronic health record (EHR) using the *International Statistical Classification of Diseases and Related Health Problems* (9th ed.) codes for TIH (852.00–853.10, 851.00–851.90, 800.00–801.9, 803.00–804.9) from January 1, 2009, through December 31, 2015. We excluded patients who had a GCS ≤ 12. Individuals with trauma to other organ systems (defined as requiring a consultation with a service other than neurosurgery) were then excluded to create a subgroup of individuals with isolated cranial trauma. We reported data as recommended by the Strengthening the Reporting of Observational Studies in Epidemiology (STROBE) statement.^17^

### Data Collection

We created a data entry form and stored the data in Microsoft Access (Microsoft Corporation, Redmond, Washington). After the initial database query of the EHR was conducted and we excluded ineligible patients (defined above), chart data were abstracted from physician notes, radiology reports, laboratory data, and discharge summaries into a standardized data collection instrument. Four trained emergency physician reviewers who were not blinded to the study hypothesis abstracted clinical data. Abstractor output was reviewed after the first 100 charts, and again at intervals throughout the review process. Reviewers met periodically to review the abstraction process; ambiguous charts were reviewed at that time. A senior investigator monitored the progress of the abstractors. Conflicting abstraction was resolved by consensus of the primary investigators after in-depth chart review. For patients discharged from the ED or the ED observation unit (EDOU), records were reviewed for any subsequent TIH-related admissions. No data were missing for any of the key clinical variables. We gave priority to real-time data over summary data. A subset of data for the clinical outcome variables was abstracted by a second, board-certified emergency physician, and inter-rater reliability was assessed using kappa statistics calculated for key variables.

Data collected included age, gender, insurance status, transferring hospital, disposition from the ED, hospital length of stay (LOS), admitting service, anti-platelet use (aspirin and other anti-platelet agents), daily anticoagulant (warfarin, novel oral anticoagulant [NOAC]) use, mechanism of injury, pre-transfer GCS, GCS at the time of initial neurosurgical evaluation, initial cranial CT results, follow-up CT results, neurosurgical exam at admission (mental status, cranial nerve exam, strength exam, sensation exam), neurosurgery recommendations (surgery including burr hole drainage, intracranial pressure monitoring, pharmacotherapy, platelet administration, anticoagulation reversal, routine repeat head CT), neurological deterioration (worsening of reported symptoms, seizures, change in neurologic examination including lethargy, somnolence, agitation, delirium, new focal deficit, seizures, worsening headache, nausea or vomiting), and neurosurgical procedures performed. Clinical historical and physical exam variables were gathered from the initial emergency medicine and neurosurgery notes.

We abstracted clinical course and information reflecting overall trauma burden (other organ systems) from hospital discharge summaries. Follow-up information after discharge was collected from visit notes from the hospitals and medical practices in the healthcare system. Cranial CT results were abstracted from the finalized attending radiologist reports. The number, location and size of hematoma(s) were noted, along with the presence of midline shift. We counted confluent hematomas as a single lesion (e.g., a frontoparietal SDH). As is routine at the study hospital, patients transferred from an outside hospital had their scans uploaded and interpreted by in-house radiologists and these interpretations were coded as the first CT finding. Radiographic data were abstracted separately using a different data form in order to blind the abstractor to the rest of the patient’s clinical information and outcomes.

Per protocol at the study hospital, all patients with TIH received a neurosurgical consultation. Patients routinely underwent repeat neuroimaging at six hours and subsequently as indicated by the treating team. Initial disposition of these patients was governed by an institutional head trauma guideline, which considers patient and scheduling factors. This first separates out patients with isolated mild head injury who are stable for monitoring in an EDOU; patients could also be placed in observation at the discretion of the emergency physician or neurosurgical attending physician. Subsequently, patients with multisystem traumatic injuries were admitted to the trauma surgery service while those with isolated non-operative head trauma (patients outside of EDOU criteria for SDH < 10mm, GCS 15) were admitted on a rotating basis to the neurosurgery, trauma surgery or neurology services. The hospital’s institutional review board approved this study.

### Analysis

We performed an initial univariate analysis looking at the association between individual factors and discharge in less than 24 hours using chi-square tests. Multivariate logistic regression analysis used variables that were significant in the univariate analysis at p ≤ 0.2. We then removed variables in a forward stepwise fashion using Statistical Package for the Social Sciences version 21 (IBM Corporation, Armonk, New York).

## RESULTS

**Figure 1:**
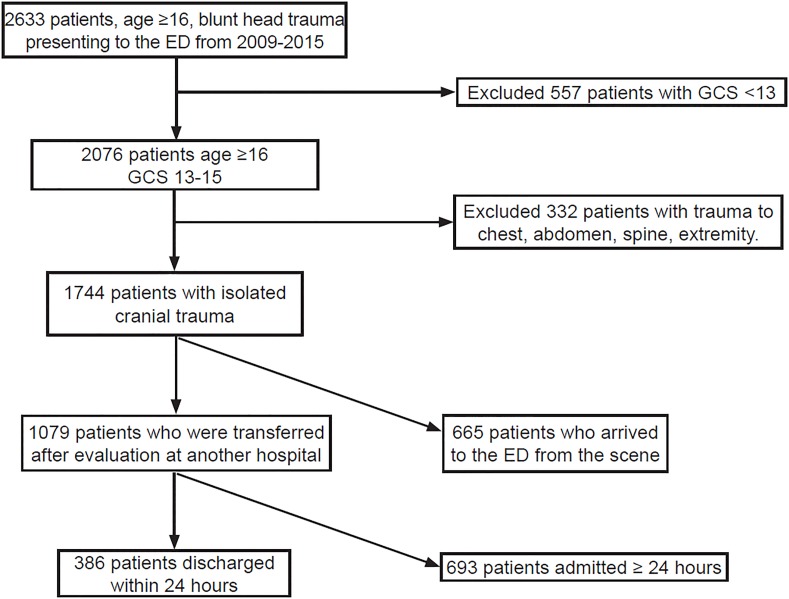
Flow chart summary of patients enrolled into the study. *ED,* emergency department; *GCS,* Glasgow Coma Scale Score.

**Figure 2:**
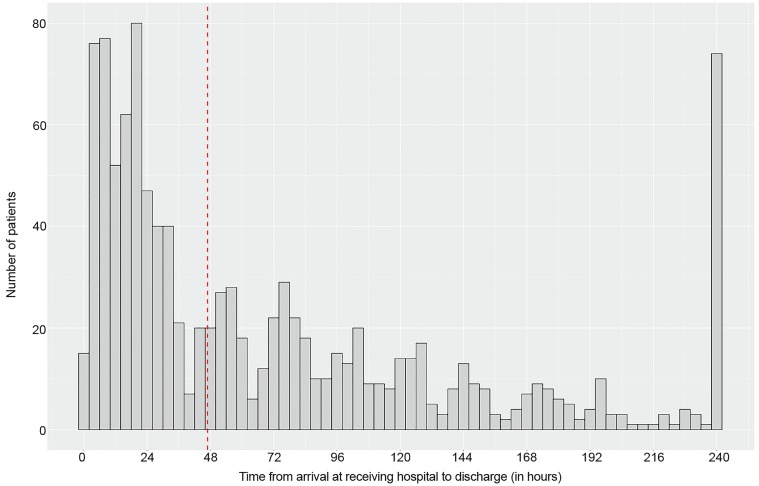
Distribution of hospital length of stay for transferred patients with traumatic intracranial hemorrhage and GCS ≥ 13. *GCS,* Glasgow Coma Scale Score. Dashed line indicates the median length of stay.

The patient selection process is summarized in Figure 1. There were 1079 patients enrolled with isolated mTIH who were transferred for neurosurgical evaluation and further treatment. Table 1 compares clinical characteristics of patients whose LOS was equal to or greater than 24 hours with the RDAT patients. Table 2 summarizes the CT findings, clinical outcomes and dispositions of these two groups. Inter-rater reliability was excellent for the clinical outcome variables. We routinely did repeat CTs to document intracranial hemorrhage (ICH) stability, and patients in the < 24-hour LOS group had a mean of 2.1 CTs (range 2–5) performed during their admission while patients who had a LOS > 24 hours had a mean number of 2.7 CTs (range 2 – 14). Patients undergoing neurosurgical intervention account for the wider range as we measured all brain imaging during the admission period including post-operative imaging. LOS in our cohort is described in Figure 2. The median LOS for this cohort was 47 hours. LOS was not significantly different when compared for each year of the seven-year study period (p=0.42). Patients with prolonged LOS represent a cohort of more complex medical conditions and debilitation that was the major contribution to their fall and subsequent ICH. Evaluation and treatment of their underlying medical issue (eg.., syncope, infection, cancer) and obtaining a safe discharge plan (rehabilitation bed or skilled nursing facility) were shared themes in these patients. Kappa values were 1.0 for need for neurosurgical intervention, 0.88 for radiologic worsening and 0.86 for the neurologic deterioration variable. Because LOS was an administrative variable, kappa values were not calculated.

The primary objective was to quantify the rate of RDAT in patients transferred with mTIH: 386 patients (35.8%) were discharged within 24 hours after transfer. Multivariate logistic regression identified three variables associated with a RDAT: GCS of 15, isolated traumatic SAH, and a SDH whose thickness was 6mm or less (Table 3). Most RDAT patients were either discharged directly from the ED or from the EDOU. Ten patients in the RDAT group received fresh-frozen plasma for vitamin K antagonist reversal. As anticipated, none of the patients discharged within 24 hours required ICU admission, intubation or a neurosurgical intervention. Additionally, 50.6% of mTIH patients were discharged within 48 hours. There were no patients who suffered a neurologic deterioration during transfer from the first to the second hospital.

Two transferred patients died within 24 hours of transfer. The first was a 92-year old-female with critical aortic stenosis and congestive heart failure (CHF) who had multiple falls and was found to have a 3-millimeter SDH and small amount of SAH. She developed respiratory distress, had a “do not resuscitate/intubate” code status and was admitted for comfort care/CHF; she passed away with respiratory failure. The second patient was a 93-year-old male with lung cancer, pulmonary fibrosis and a pacemaker who fell and struck his head. He had subarachnoid blood on his CT. He had pacer malfunction with complete heart block, and given his “do not resuscitate” status and a discussion with his family he was made comfortable, admitted, and then passed away.

Follow-up information was available in 85.3% (with 83% of follow-up occurring greater than 30 days after the index visit) of all patients and 76% of patients discharged from the ED or EDOU (with 87% of follow-up occurring greater than 30 days after the index visit). There were no noted deaths within 30 days. Eight patients returned to the ED with a complication related to their intracranial hemorrhage, and five patients were admitted. Three patients had delayed, planned neurosurgical procedures after discharge. These cases are further detailed in the Appendix.

## DISCUSSION

mTIH is a relatively common diagnosis and, while there is ample evidence showing that mTIH is primarily treated non-operatively,^2^,^3^,^11^ the remainder of management of these patients, including which subgroups need repeat imaging, ICU monitoring and transfer to tertiary trauma centers, remains unclear. Given the lack of clear, evidence-based guidelines, management of patients with mTIH may present an interesting opportunity to optimize resources. This study takes the first step toward creating a framework for transferring head-injured patients by examining the outcomes of patients transferred to a single Level I trauma center, along with factors associated with RDAT.

RDAT was common after transfer of patients with mTIH, occurring in greater than one-third of transferred patients. While this is the first study to evaluate the transfer of patients with mTIH, other studies examining the utility of trauma transfer have shown similar rates of expedited discharge, ranging from 6% to 39%.^14^,^15^,^18^ These data reflect the fact that mTIH patients behave similarly to other mildly injured trauma patients, and that TIH alone without change in mental status does not pose an increased risk compared to other trauma patients. Additionally, the high rate of rapid discharge, coupled with the fact that only 1% of patients returned with delayed or missed injuries, indicates that a carefully identified cohort of these patients might be able to be managed in a community setting.

Patients with traumatic SAH (tSAH) are at particularly low risk. It has been suggested that tSAH is a benign entity that can be treated as a concussion.^19^ The same conclusion was reached in another series of 120 tSAH patients admitted to an EDOU.^2^ This subject has also been the focus of a recent meta-analysis of over 15,000 tSAH patients.^20^ Since tSAH are not expansile surgical lesions, it is expected that patients with isolated tSAH would have decreased need for intervention when compared to SDH, which can exert pressure and cause the brain to shift.

It is surprising that coagulopathies were not prominently represented as a high-risk variable in this report. Many patients who were on warfarin had attempted reversal with vitamin K and fresh frozen plasma (FFP) prior to transfer. Additional FFP was used in the ED if the international normalized ratio had not decreased to less than 1.4. During this study period, it was also a standard neurosurgical recommendation to transfuse dose of platelets for any patient on anti-platelet drugs including aspirin. These interventions may explain why there were not more negative outcomes in coagulopathic patients. It may be that CT stability after complete reversal doesn’t impart any more risk than in a patient who never had abnormal bleeding parameters. Our study had few patients on NOACs, so conclusion and comments on the effects of these drugs are beyond the scope of this paper.

This idea of delayed transfer of patients with mTIH is not a new one and came out of necessity given distances between some hospitals and the closest regional trauma center. Levy reports on a non-transfer protocol for patients with GCS 13–15 and with available consultation and CT image review with a neurosurgeon at a nearby Level I trauma center.^21^ An Israeli study of three trauma centers without neurosurgical backup demonstrated that teleconsultation and clear, imaging-specific transfer protocols could drastically reduce transfer frequency.^22^ Finally, Rhee and colleagues demonstrated the safety an mTIH protocol that included a six-hour observation period without neurosurgical consult or routine, repeat head CT.^23^

There are several barriers to overcome before patients with mTIH can be cared for at a hospital without neurosurgical capabilities. Researchers must gather prospective evidence examining the propensity of different lesion types to cause neurologic deterioration and the ability of non-neurosurgical providers to manage these lesions.^24^–^26^ If a decision is made to trial a non-transfer protocol it makes greater sense to do so in an urban area, where transport times to a neurosurgeon are low. There would need to be a decision made regarding which service/provider (EDOU, hospitalist, neurologist, acute care surgeon) would care for these patients. A protocol^27^ should be in place to allow a rapid accept/transfer mechanism to a Level I trauma center to ensure timely intervention or ICU level of care. Telemedicine may be used to provide experienced backup. Finally, patients will need follow-up to monitor resolution of ICH and neurologic complaints. Neurosurgical consultation and follow-up is recommended in cases of persistent ICH or neurologic symptoms and in patients who need to be restarted on anticoagulation or antiplatelet therapy.

## LIMITATIONS

The most important limitation of this investigation was that it was performed at a single institution with substantial neurosurgical expertise and ED providers who are accustomed to evaluating and monitoring brain-injured patients. Additionally, all patients were evaluated by neurosurgeons during their ED stay. While given the paucity of interventions performed, it is unlikely that these evaluations changed outcomes and it is difficult to apply these findings toward transfer practice at community hospitals, limiting the generalizability of the results. Additionally, transfer rates for mTIH may be different nationally than within the single trauma system studied here. Unfortunately, national estimates of trauma transfer rates are not available, and the highly region-dependent nature of trauma care would limit even national estimates. However, this investigation still highlights an area for potential improvement, and the risk factors identified here are broadly applicable.

As this was a retrospective study, there were some intrinsic data validity issues, but the study design attempted to minimize these. The variables included in the study were either administrative or not subject to significant interpretation as indicated by kappa levels of our primary clinical-outcome variables. Due to resource constraints, CTs could not be re-read by a radiologist blinded to the clinical status of the patient. Instead, the final CT interpretations approved by the attending radiologist were used. Some patients who were discharged from the ED may have been initially intended to have been placed in the EDOU or admitted but were instead directly discharged from the ED after a period of observation due to bed availability. There was no way to retrospectively determine this intent from the EHR/administrative record. Additionally, the criteria for when to perform a neurosurgical intervention are not well defined in patients who are neurologically intact, an issue that no doubt influenced our neurosurgical intervention variable. All of these issues highlight the importance of future prospective examinations of patients with mTIH, so that these factors can be appropriately accounted for in the study design.

Because it was not possible to gather data from the sending facility, the first available examination was the initial evaluation at the tertiary care site. Therefore, there is some possibility that patients’ clinical status may have changed substantially during the period of transfer, leading to the exclusion of some patients who deteriorated on transfer. While this could not be directly addressed in this analysis, the fact that patients who presented directly from the scene had similar clinical outcomes to the transferred group suggests that this phenomenon probably did not have a significant effect. Again, this suggests the importance of a future, multicenter prospective study.

Finally, even though our follow-up approached 80%, the development of a complication after head trauma can be a rare event and it is possible that patients lost to follow-up suffered a neurologic event and either died or were taken to a hospital outside of our network.

## CONCLUSION

Our investigation showed that rapid discharge after transfer was a common phenomenon, occurring in greater than one-third of patients with mTIH who were transferred to a tertiary care center. Further prospective, systems-based research should attempt to determine a low-risk subgroup of these patients and create systems that allow them to be cared for in the community.

## Supplementary Material



## Figures and Tables

**Table 1:** Patients discharged within 24 hours after interfacility transfer when compared to patients with longer length of stay clinical variables.

	Discharged within 24 hours N=386	LOS ≥ 24 hours N=693	Comparison		P value
		
	%	%	OR	95 CI	
Demographic/history					
Age≥60	54.1	72.6	2.2	1.7–2.9	< 0.0001
Male sex	55.2	56.1	1.0	0.8–1.3	0.7
Aspirin use	17.1	28.7	1.9	0.1–2.7	< 0.0001
Warfarin use	4.2	12.4	3.3	1.9–5.7	< 0.0001
Other Anti-platelet	2.6	4.8	1.9	0.9–3.9	0.08
NOAC	0.3	0.1	0.6	.03–8.9	1.0*
HTN	40.2	48.1	1.4	1.1–1.8	0.01
Intoxicant	17.6	14.1	0.8	0.5–1.1	0.13
Insurance					
Private	52.3	35.4	0.5	0.4–.6	< 0.0001
Medicare	36.8	53.9	2.0	1.6–2.6	< 0.0001
Medicaid	6.5	6.6	1.03	0.6–1.7	0.9
Self-pay	4.4	4.0	0.9	0.5–1.7	0.7
Mechanism					
Fall	72.3	83.1	1.9	1.4–2.5	< 0.0001
MVC	7.3	4.3	0.6	0.3–.9	0.04
Assault	13.2	6.2	0.4	0.3–.7	< 0.0001
Pedestrian struck	0.8	1.4	1.9	0.5–6.8	0.39*
Bicyclist struck	4.2	1.6	0.4	0.2–.8	0.009
Motorcycle collision	1.6	1.6	1.0	0.4–2.8	1.0
GCS					
15	91.5	77.5	0.32	0.2–.5	< 0.0001
14	7.5	16.2	2.4	1.5–3.6	< 0.0001
13	1.1	6.4	6.5	2.3–18	< 0.0001
Clinical outcomes					
Neurologic event	1.0	8.9	9.4	3.4–26	< 0.0001
Repeat CT worse	2.9	10.1	3.8	2–7.3	< 0.0001
Neurosurgical intervention	0	11.8	n/a	n/a	< 0.0001^f^
Death	0.5	2.9	5.7	1.3–24.5	< 0.0001

*LOS,* length of stay; *OR,* odds ratio; *CI,* confidence interval; *NOAC,* non-vitamin K antagonist oral anticoagulants; *HTN,* hypertension; *MVC,* motor vehicle collision; *GCS,* Glasgow Coma Scale Score; *CT,* computed tomography.

* P value in some cases is significant for discharge within 24 hours, and in other cases for >24 hours.

f Fisher exact test.

**Table 2:** Patients discharged within 24 hours after interfacility transfer when compared to patients with longer length of stay: radiologic findings and disposition variables.

	Discharged within 24 hours N=386	LOS ≥ 24 hours N=693	Comparison		P value
		
	%	%	OR	95% CI	
CT lesions (all)					
Any SAH	45.6	48.2	1.1	0.9–1.4	0.4
Any SDH	52.6	66.2	1.8	1.4–2.3	< 0.0001
Any EDH	2.9	3.9	1.4	0.7–2.8	0.4
Any contusion	22.0	28.0	1.4	1.0–1.8	0.03
Any skull fracture	14.3	15.3	1.1	0.8–1.5	0.6
CT lesions isolated					
Isolated SAH	25.9	17.0	0.6	0.4–0.8	< 0.0001
Isolated SDH	34.5	36.2	1.1	0.8–1.4	0.5
Isolated EDH	0.5	0	1.0^f^		1.0^f^
Isolated contusion	9.1	7.7	0.8	0.5–1.3	0.5
Isolated skull fracture	4.2	2.2	0.06	0.2–1	0.06
Depressed skull fracture	0.5	0.6	1.1	0.2–6	1.0^f^
SDH ≥ 6mm	12.7	26.0	2.4	1.7–3.4	< 0.0001
SDH ≥ 10 mm	5.2	19.0	4.3	2.6–7	< 0.0001
Any SDH midline shift	5.4	16.7	3.5	2.2–5.7	
Disposition					
ICU admission	0	19.1	<0.0001	2.8–5.0	< 0.0001
Floor admission	19.2	60.6	3.7	0.3–0.5	< 0.0001
EDOU	38.1	18.9	0.4	0.01–0.04	< 0.0001
Treated/released	41.7	1.4	0.02		< 0.0001
AMA/LWCT	1.0	0	0.02^f^		0.02
Admitting services					
Trauma	6.0	20.4	4.0	2.5–6.4.0	< 0.0001
Neurosurgery	3.4	24.9	9.5	5.3–17.0	< 0.0001
Neurology	7.8	22.8	3.5	2.5–5.3	< 0.0001
Medicine	1.6	11.1	7.9	3.4–18.3	< 0.0001
Pediatrics	0.5	0.4	0.8	0.1–5.0	1.0^f^
Emergency/EDOU	38.1	18.9	0.4	0.3–0.5	< 0.0001
Emergency/discharged	41.7	1.4	0.02	0.01–0.04	< 0.0001
AMA/LWCT	1.0	0	0.02^f^		0.02^f^

*LOS,* length of stay; *OR,* odds ratio; *CI,* confidence interval; *CT,* computed tomography; *SAH,* subarachnoid hemorrhage; *SDH,* subdural hematoma; *EDH,* epidural hemorrhage; *ICU,* intensive care unit; *EDOU,* emergency department observation unit; *AMA,* against medical advice; *LWCT,* left without completing treatment.

P value in some cases is significant for discharge within 24 hours, and in other cases for >24 hours.

f Fisher’s exact test.

**Table 3:** Multivariate logistic regression: variables associated with length of stay < 24 hours.

Variable	OR	95% CI
GCS 15	2.9	1.9 – 4.4
SDH 6mm or smaller	3.1	2.2 – 4.5
Isolated SAH	1.7	1.3 – 2.4

*OR,* odds ratio; *CI,* confidence interval; *GCS,* Glasgow Coma Scale Score; *SDH,* subdural hematoma;* SAH,* subarachnoid hemorrhage.
