# Supplementary material for: Rapid Discharge After Interfacility Transfer for Mild Traumatic Intracranial Hemorrhage: Frequency and Associated Factors
Source: West J Emerg Med. 2019 Feb 11;20(2):307–15. doi: 10.5811/westjem.2018.12.39337 (PMC6404693; doi:10.5811/westjem.2018.12.39337)
Supplement: Supplementary file 1 [file wjem-20-307-s001.docx]

Appendix Table 1. – Follow up of Patients Treated and Released from the ED and EDOU as well as patients admitted from EDOU to an inpatient service

| Patients treated and release from ED after transfer with mTIH related problems | |
| --- | --- |
|  |  |
| Case 1 | 40F no coagulopathy with fall, had SDH 10mm. 5 days later had nausea and vomiting. A follow up scan revealed 13mm SDH and had burr hole procedure |
| Case 2 | 88M fall, no coagulopathy had 2mm SDH but 2 weeks later had second fall. Evaluated 2 weeks after that had 28mm SDH and underwent Burr hole procedure |
| Case 3 | 85M fall no coagulopathy with fall SDH 18 mm chronic SDH. Had a return visit for headache and had a stable repeat CT in ED discharged back to rehabilitation facility |
|  |  |
| Patients discharged from EDOU after transfer with mTIH related issues | |
| No. of patients | Post discharge complications |
| 18 | Return for evaluation, concussive symptoms, repeat CT unchanged or improved |
| 1 | 58F, fall no coagulopathy. Patient with post traumatic seizure 18 days later, CT unrevealing |
| 1 | 70M, s/p fall Developed worsening headache and readmitted for acute on chronic SDH after restarting aspirin 5 weeks later and had no intervention. |
| 1 | 87F, had fall and SDH. Developed right middle cerebral artery stroke 5 weeks later. Had been taken off aspirin |
| 1 | 52F no coagulopathy, had a fall, persistent headache and chronic SDH .Underwent burr hole drainage 3months later |
| 1 | 90F no coagulopathy had fall had cerebral contusion. Reimaged for headache and found to have chronic SDH. No interventions |
| Patients admitted from the EDOU to inpatient service | |
| No. of patients | Reason for admission |
| 15 | More time was needed for physical therapy, rehabilitation bed search |
| 8 | Medical conditions requiring admission, urinary tract infection, chest pain, cancer workup |
| 4 | Persistent concussive symptoms and episodes of delirium |
| 3 | Monitoring of possible seizure |
| 2 | Increased size or new lesion seen on CT scan |
| 2 | Burr Hole Surgery |

Abbreviations: SDH – subdural hematoma, mTIH – mild traumatic intracranial hemorrhage, EDOU – ED observation unit
